# Microbial Biofungicides as a Substitute for Chemical Fungicides in the Control of Phytopathogens: Current Perspectives and Research Directions

**DOI:** 10.1155/2024/5322696

**Published:** 2024-02-28

**Authors:** Lamenew Fenta, Habtamu Mekonnen

**Affiliations:** ^1^Department of Biology, Debre Markos University, Debre Markos, Ethiopia; ^2^Department of Biology, Bahir Dar University, Bahir Dar, Ethiopia

## Abstract

These days, two important issues are causing concern in the global community: the alarmingly growing trend of the human population and the issue of food security. To this end, people around the world have been searching for solutions that could feed the needy in a sustainable way. In response to this urgent call, scientists from around the world started working on increasing crop production and productivity by controlling crop pathogens that could harm the productivity of crops. Synthetic fungicides have been in use for controlling crop diseases for several decades, but later, due to the evidenced side effects of the fungicides, there have been attempts to shift towards a less cost-effective and eco-friendly method of controlling crop diseases, and so far, many remarkable results have been achieved. However, due to the less effective and shorter shelf life of microbial biofungicides, as well as the less accessibility of these microbial biofungicides to growers around the world, it became difficult to remove the fungicides totally from the market. To minimize this problem, researchers suggested an integrated approach: the combination of microbial biofungicides with a reduced dose of synthetic fungicides. Hence, this review explored the status as well as the merits and demerits of microbial biofungicides as compared to synthetic fungicides.

## 1. Introduction

Global food security is one of the major issues that needs the utmost attention of the scientific community in the near future. The growing food demand of the society is putting enormous pressure on the resources over which the food supply of the civilization depends. The world's food production has to double in order to keep up with the rate of population growth. However, the influence of plant pathogens on the loss and productivity of major crops is increasing, and this challenge is more pronounced in developing countries [1]. Many plant pathogens cause diseases in agricultural fields [2]. They can range from viroids of a few hundred nucleotides to higher plants. Their results range from mild symptoms to disasters in which vast areas are devastated by food crops. Over 800 million people worldwide lack access to enough food; 1.3 billion people survive on less than $1 per day; and at least 10% of the world's food production is lost to deadly plant diseases [3].

In recent decades, efforts are being taken all over the world to increase food production. This is achieved through the development of improved, disease-resistant varieties of staple crops; the increased use of chemical fertilizers and pesticides; and the expansion of irrigated cropland. However, these efforts did not seem to be quite fruitful as the rate of population growth in certain areas was much higher and their increased food production could not cope with the increasing population pressure [1]. Now, the challenge is to feed more with less environmental damage. So, taking urgent measurements on plant pathogens that cause huge damage and loss is a top priority for concerned bodies. Adoption of technologically sound, traditional knowledge-inclusive, socioeconomically sensible recommended agricultural practices can be the basis for achieving future food demands [4].

Sustainable agriculture is necessary for maintaining farmer livelihoods, enhancing food and nutrition security, and sustaining long-term national growth [5]. The improvement or maintenance of environmental quality while simultaneously protecting natural resources is a prerequisite for sustainable development [6]. Thus, sustainable agriculture necessitates the efficient management of agricultural resources in order to control pathogen and disease issues to the point where they do not negatively influence crops by upsetting the natural balance [7]. Synthetic fungicides have been used to control crop diseases and increase crop production for many years [8]. Although synthetic fungicides reduce the loss of crops, excessive use of synthetic fungicides has resulted in pathogenicity resistance, pathogen resurgence, and pathogen extinction. They are also harmful to aquatic life, soil biodiversity, humans, and animals [9]. Typical effects of these fungicides include soil embrittlement, decreased soil respiration, and decreased activity of several soil microorganisms [10]. Synthetic fungicides reduce animal vitality, immunity, and the efficacy of animal reproduction [11]. Synthetic fungicides have a detrimental effect on plant growth by reducing the biological function of soil microbes in producing specific plant growth-promoting properties such as indole-3-acetic acid, nitrogen, and siderophores [12]. Fungicide spills can enter water bodies and cause pollution and the destruction of aquatic life. Moreover, fungicide bioaccumulation in aquatic settings has been linked to the development of deadly diseases such as diabetes, rashes, kidney disease, and cancer in both animals and people as well as aquatic organisms [13].

The EU and US have already outlawed some chemical pesticides due to their detrimental effects, and many conventional items have also been phased out due to concerns over their effects on the environment and human health [14]. The limited biodegradability and high persistence of synthetic pesticides are some major drawbacks [15]. Contamination of the environment (water and soil), deleterious effects of fungicide residues on beneficial insects (earthworms, bees, and spiders), and detrimental effects on soil microbiota result in biodiversity loss and disturbances in the cycle of nutrients [16]. In these situations, an integrated strategy provides a variety of management options that are sustainable and benign to the environment while still protecting human and environmental health. Biological control, which employs effective biocontrol agents to lessen pest damage, is a key component of a comprehensive strategy [17]. The primary idea is to employ microbes and their products to control the plant diseases without affecting features and elements of the ecological environment [18]. In the era of sustainable agriculture, microbial biofungicides provide a solution for issues including fungicide resistance, environmental concerns, and human health issues [19].

Biofungicides are believed to be significantly more environmentally friendly than natural fungicides, yet this long-term option is vying for attention in the present synthetic pathogen market. The key issues relating to technological difficulties and long-term sustainability require an urgent need of resolution for more adaptability to popularize or promote the microbial biofungicides. These microbial biofungicides may take the form of microbial fungicides (microbial origin) [20], phytofungicides (plant origin) [13], and nano-biofungicides (nanoparticles manufactured from biological substances) [21]. Microbial biofungicides are less expensive, more accessible, and long-lasting than synthetic fungicides, and also, they have no unwanted effects unlike synthetic fungicides [22]. Phytofungicides, in addition to possessing a diversity of phytochemical components that give them different modes of action, are less hazardous to human health than synthetic fungicides [23]. Nanobiofungicides outperform synthetic fungicides in terms of fungicidal action, controlled or targeted release, biodegradability, and good biocompatibility [21]. Therefore, the major goal of this review is to assess the progress of microbial fungicides, their potential to replace chemical fungicides, their drawbacks, and to suggest a basis for future research that will be most helpful in managing phytopathogens. The present review also discusses the effects of using synthetic fungicides to manage crop pathogens and to explore the role of microbial biofungicides in the management of plant diseases and to outline the current trends and status of utilizing these mechanisms.

## 2. Microbial Biofungicides

**Figure 1:**
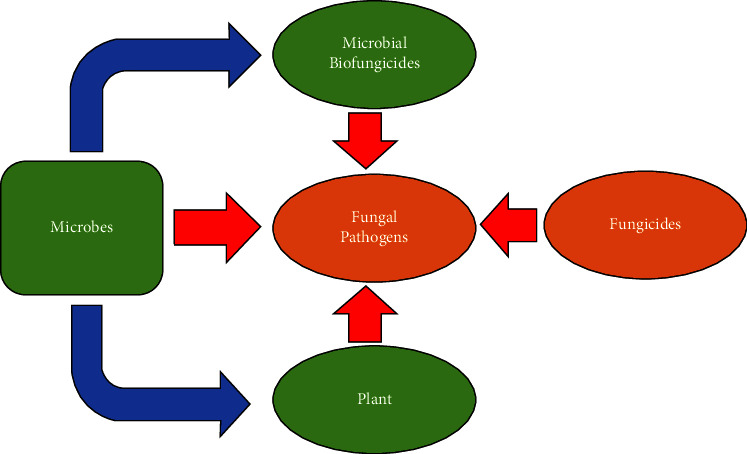
Microorganisms as an alternative to conventional fungicides.

**Figure 2:**
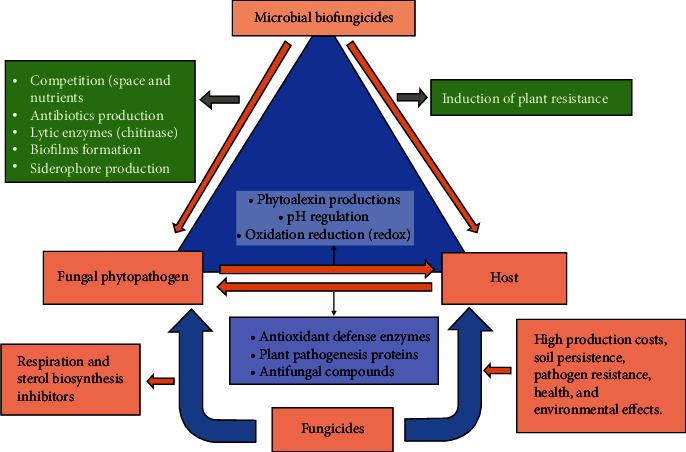
Mechanisms of action of microbial biofungicides and conventional fungicides against fungal phytopathogens.

In the recent times, the utilization of microbial biofungicides is catching up the attention of many researchers because of their less toxic effect and lower cost. Microbial biofungicides are capable of inhibiting a wide variety of infections, and each active component is tailored specifically to a pathogen that has to be controlled while being safe for other organisms (Figure 1) [13]. These fungicides can be supplied as spores, living organisms, or dead organisms, and they are typically sprayed on crops in the same manner as chemical fungicides. Due to their target specificity, repeatability, and ability to provide ongoing disease control, the active components that have potential benefits over chemical fungicides are higher because they are living organisms [24]. Plant pathogens are suppressed by these microbial biofungicides because they prevent the growth of competing organisms, which in turn causes disease and produces specialised toxins [23]. Microbial biofungicides are a sought-after component for integrated pathogen management because of their unique and varied range of features. The main mechanisms of action exerted by microbial biofungicides are competition for space and nutrients, suppression via siderophores, hydrolytic enzymes, antibiosis, biofilm formation, and induction of plant resistance, while the most common fungicide modes of action are respiration inhibitors and sterol biosynthesis inhibitors [25, 26] (Figure 2).

Microbial biofungicides normally have less adverse impacts on the environment, agricultural product producers, or consumers due to their target-specific nature and generally safe ingredients [27]. Also, when compared to chemical fungicides, their use results in lower greenhouse gas emissions [13]. Moreover, a wide range of organisms can be used to produce microbial biofungicides, which can be sustainable and can tackle the issue of resistance. As different bacteria utilized as microbial biofungicides may require different storage conditions and because of difficulties with in-depth scientific research, ecological studies, and mass production methods, we have a limited understanding of microbial fungicides [28]. Dealing with its storage and transit may be challenging for sellers, producers, marketers, and end users. Thus, more study is required to guarantee a long shelf life for microbial fungicides.

Many techniques are utilized to apply microbial biofungicides, including spray drying, spray chilling, lyophilization, coacervation, fluidized beds, extrusion, and electrospraying [29]. The two main types of microorganisms that are employed as microbial biofungicides are bacteria and fungi. Many fungi that live in the soil and cause different plant decays have been shown to be inhibited by bacterial genera including *Pseudomonas*, *Bacillus*, *Yersinia* [30], and *Trichoderma* spp. [31] and other bacteria.

### 2.1. Challenges of Microbial Biofungicides

Even though they offer promising futures in the management of plant pathogens, there are restrictions on the usage and efficacies of microbial biofungicides. Some of the challenges are addressed below.

#### 2.1.1. Product Development and Formulation

Many researchers have had great success using microbial biofungicides in the lab, particularly with noncommercial biological agents. The move from the lab to the outdoors, however, has not been very successful. The reports for the transfer of biofungicide from the laboratory to the field have been difficult [32]. The challenge is a result of product development and formulation. To keep biocontrol agents (BCAs) alive, effective, and useable as intended, special formulation and storage techniques are required [33]. Biocontrol agents distinguish themselves from other types of control agents because they are living organisms. Due to their higher sensitivity to microclimate, they may also need special treatment during storage, shipment, and use in addition to their formulation requirements [34]. Inoculum concentrations in microbial biofungicides, especially microbial biofungicides, present another problem in product formulation. Most tests revealed that various variables both *in vivo* and *in vitro* can vary. For instance, flower infection was not prevented by treating any of the BCAs at 10^6^ conidia/mL at 15°C, the typical temperature in the field conditions. Nevertheless, 10^6^ conidia/ml at 25°C was successful *in vitro*. However, doubling the concentrations (to 10^8^ conidia/ml) at 15°C prevented flower infection [35]. Similar findings were made by Kim et al. [36], who found that greater antagonist doses (10^8^ CFU/mL) improved tomato gray mold biocontrol.

The impact of the production process on the product's viability is another difficulty in the creation of biological control products. It has been discovered that culture conditions, including conidial age and production temperature, have an impact on BCA germination and bioactivity. For instance, *Trichoderma atroviride* reached its maximum growth potential at 25°C, but the maximum germination and bioactivity were found in conidia generated at 30°C. This implies that cultural conditions have an impact on the formulation of biological controls. Several naturally occurring substances derived from plants and microorganisms are typically found in low concentrations and are challenging to purify on a large-scale basis [37]. The absence of standardized extraction techniques is one of the main problems with microbes and plant-based natural compounds. The various extraction techniques are probably going to affect disease control goods differently, which will ultimately affect how effective these medicines are. Agrochemical businesses create innovative chemicals and semisynthetic derivatives from these natural substances due to the difficulty of creating natural commercial products. Natural substances are quite helpful, but their significance cannot be emphasized if processes are not standardized to provide consistent and repeatable results [38].

#### 2.1.2. Developing a Product for a Pathogen that Affects Several Hosts

The difficulty of biologically managing phytopathogens includes product development. The optimum response to widespread and multihost infections is to provide a treatment that can be used on cropping systems and all hosts, such as most synthetic fungicides. It is challenging to create solutions that are effective across a variety of hosts and geographical locations due to the complexity of the virus and its varied interactions with biocontrol agents and animals. It is extremely challenging to develop a biocontrol product that can successfully survive and provide sustainable disease control under these varying settings given the nonspecialized nature of phytopathogens and their adaptability to varied hosts, environments, and to some extent, cropping systems [39]. Finding biocontrol strains that are well-suited to hosts and farming systems might have implications for disease management.

#### 2.1.3. Inconsistency on the Field

The use of this approach has been severely impeded by the unreliability of microbial biofungicides in the field. Although microbial biofungicides have achieved considerable achievements in lab and greenhouse settings, several of them do not consistently control disease when used in the field [40]. There could be several reasons for the inconsistencies and decreased efficacy of microbial biofungicides that have been observed in real-world settings.

#### 2.1.4. Effects of Environmental Variables on Microbial Biofungicides

The ability of biocontrol agents to adapt to different climatic and environmental settings, as well as evidence of considerable efficiency against the target disease in a variety of scenarios, is an essential factor that contributes to their success in both greenhouse and field conditions. Temperature, relative humidity, and UV rays are all elements that affect the lifespan of biocontrol agents [41]. These circumstances offer a diversified microbiota with bacteria tailored to a particular environment. Microbes can manifest themselves differently from year to year as well as at various sites. These influences may be effectively managed in greenhouses to increase BCA survival. It still needs to be completely addressed how to keep greenhouse conditions that simultaneously suit the needs of both biocontrol agents and crops. BCAs and organic materials used in the field are regularly exposed to a variety of temperatures and relative humidity. The efficacy of biocontrol techniques is substantially hampered by the mismatch between disease environmental requirements and BCAs. For instance, *Botrytis cinerea* is active throughout a wide temperature range, with an optimal range of 15–20°C [42], whereas the ideal temperature for most *Trichoderma* species usually employed to control *B*. *cinerea* is 25–30°C [43] and 20–25°C for *Bacillus* species [44]. It is quite likely that *B. cinerea* will quickly colonize space at temperatures below 20°C given its rapid colony proliferation and conidia generation under biological control with the BCA in the field. This will have a considerable impact on how well these biocontrol agents work, especially those such as *Ulocladium* spp. and *Trichoderma* spp. that compete with one another for nutrients and space. Temperature and relative humidity can be effectively managed in greenhouses, but due to the variety of the indoor microclimate and the uniqueness of each greenhouse, BCAs are likely to have a varied level of efficiency when compared to synthetic fungicides. Due to the stark differences between BCAs and phytopathogens in terms of their environmental requirements, as well as the specifics of greenhouses and geographic locations, it is extremely challenging to develop a biocontrol product that is applicable for greenhouse or field application to various geographic locations. To overcome some of these problems with BCAs, a blend of several BCAs and an adequate high conidia concentration must be utilized.

#### 2.1.5. Application Duration and Cross-Compatibility with Other Products

Microbial biofungicides are only preventative and cannot “cure” already-infected crops [45]. As a result, knowledge-intensive management is needed for the effective deployment of BCAs. Knowing the pathogen's biology can help in disease management by determining when and where biocontrol should be used. It was discovered that the best time to apply a biocontrol product depends on the timing of the application [46]. To effectively manage disease, it is recommended to combine various biocontrol agents or use synthetic fungicides. Therefore, it is crucial to comprehend how microbial biofungicides interact with other elements and synthetic fungicides of the production process to develop practical disease control plans.

### 2.2. Synthetic Fungicides

Fungicides, despite certain limitations, continue to play a crucial role in the management of plant diseases. In their history of more than a century, several fungicide classes have been introduced starting from multisite inorganic salts to organic compounds with protectant action and then to single-site systemic fungicides with curative activity [47]. Historical perspectives on using chemicals for plant disease control include the application of effective methods for controlling plant diseases. Although IDM is recommended, synthetic fungicides remain the most important means of controlling the pathogen, and in some cases, the only option. Direct protection using synthetic chemicals is one of the basic principles of plant disease management. Fungicides, bactericides, and nematicides are applied through different methods such as foliar, slurry, drench, and paste. Fungicides can be classified based on the mode of action, usage, and composition. Limitations of pesticide usage occur in plant disease management, due to health hazards and pesticide impact on the environment. Insurgence of fungicidal resistance in plant pathogens is also a significant threat. The efficacy of chemical compounds is also affected by climate changes [48].

Recent trends in the development and use of synthetic chemicals in plant disease control consider a comparison between pesticides and alternative plant disease control methods, fungicide marketing policies, and procedures. Until recently, the use of synthetic fungicides for plant protection was thought to be safe. However, it was reported that its continuous use faces three major challenges, namely, (1) increased public concern about contamination of fruits and vegetables with residues from synthetic fungicides and its effect on human health [49], (2) increased resistance development in pathogen populations [50], and (3) environmental pollution [51].

### 2.3. Drawbacks of Synthetic Fungicides

Many crops are lost to infections every year, but losses have decreased because of the development of synthetic fungicides. Today's synthetic fungicides do, however, come with drawbacks, including high acquisition and production costs, persistence in soil, pathogen resistance, health and environmental effects, financial loss to organic producers due to pathogen migration, destruction of infected crops, disposal of expired products, and disposal of leftover fungicides and conventional tank stocks, which can harm organic farms or the public [52] (Figure 2). Several fungicides do not decompose when applied to soil for agricultural purposes. As a result, they persist longer in the ecosystem and seep into groundwater and surface waters, causing pollution and biodiversity loss. Most fungicides that are sprayed on soil influence species other than the ones they were designed to kill. Furthermore, another method by which fungicides have been linked to having a negative effect on soil nutrients is by chelating some important metal ions, which leaves them unavailable to plants [53]. Fungicides can also hinder photosynthesis, reproduction, and seed formation in plants [54].

Humans can consume the leftovers of fungal spores that affect edible plants directly, or they can be utilized to make fodder [55]. This may be relevant if fungicides are applied during harvest [56]. Three fungicides, glyphosate, malathion, and alpha-cypermethrin, were found to reduce the activity and population of fungus, actinomycetes, and bacteria in soil [57]. Animal biodiversity and genetic conservation are reduced because of all the harmful effects of synthetic fungicides. Moreover, it affects soil microbial activity. This alters soil biodiversity and health. Humans may contract several ailments if they consume milk, meat, vegetables, edible plants, fruits, or vegetables with high levels of harmful pesticide residues [58]. According to Onwujiogu et al. [59], Bambara groundnut contains fungicides that are over the WHO's recommended maximum residue levels (MRLs) and may be damaging to the health of people, especially if they are consumed by children. Moreover, testing of the elimination of fungicide levels in the three fruits showed that the pesticide level in watermelon was above the WHO/FAO residue limit, which is dangerous to consumer health [59]. Fungicides are also employed to safeguard harvested food crops, including fruits, vegetables, and grains, as well as those utilized for uses aside from those for which they were intended. For instance, using calcium carbide to ripen fruit puts human health at risk. When calcium carbide, which contains calcium phosphide and calcium arsenite, combines with water to create phosphide and arsine, it causes fatigue, headaches, nausea, vomiting, and dizziness [60]. Similarly, when tested on albino rats, the pathogen ethephon which has the ability to accelerate the ripening of vegetables, fruits, and grains showed hepatocyte characteristics [61]. Aside from these conditions, biomagnification of fungi through exposure to skin pores (during spraying), postharvest storage, food (such as fish), water, and inhalation results in conditions such as Alzheimer's disease, birth defects, cancer, cardiovascular diseases, diabetes, eczema, eye irritation, hormonal disorders, hypertension, kidney disease, liver dysfunction, neurological degeneration, Parkinson's disease, and rashes [62, 63]. Moreover, high fungicide levels have been linked to a 25–30% rise in psychological health issues and a 50% rise in relentless leukemia, lymphoma, brain cancer, and other cancers.

### 2.4. Can Microbial Biofungicides Fully Substitute Synthetic Fungicides in the Current Scenario?

The need for novel fungicide alternatives that are better for the environment and human health and could lead to the production of safer food is currently the subject of intense scientific inquiry. Despite its many shortcomings and growing concerns from farmers and consumers worldwide, alternative means of controlling the disease are being pushed forward [13, 64]. There is no complete replacement for chemical disinfectants with microbial biodisinfectants. First, microbial biofungicides themselves have many drawbacks for full onsite application and, moreover, are available in markets where chemical fungicides are still dominant and used in most agricultural systems. Yet, it might be difficult and expensive to find commercial biofungicide products on the open market. Especially in developing countries, it is almost impossible to completely replace the use of synthetic fungicides and eliminate them from the market because there is no good technology for research, commercialization, and business. Moreover, given that microbial biofungicides are not abundant on the market and have their own limitations, withdrawing synthetic fungicides from the market is not a good advice for the fungicide industry. An alternative is to use microbial biofungicides to reduce size and dose to supplement synthetic fungicides.

The applicability and the matter of commercialization have both been the subject of numerous studies. Moreover, microbial biofungicides experience issues with quality control and have a limited shelf life [65]. The recommended dosages and the assessment of potential new pathogen species that may be resistant to the current microbial biofungicides are other issues that many farmers worry about [66]. To combat plant diseases, direct-acting microbial antagonists have reportedly been coupled with synthetic fungicides. The combination of fungicides and compatible microbial biofungicides in integrated disease management (IDM) strategies not only protects seeds and seedlings from soil and seed-derived inoculum [67] but also controls the disease. It can also improve the effectiveness and provide better protection. Combinations of microbial biofungicides and fungicides may provide disease control such as increased doses of fungicides. Combining microbial biofungicides with synthetic chemicals eliminates the possibility of developing resistance and reduces the use of fungicides. For instance, combining conventional fungicides against preharvest infections with fungal antagonists improves disease management. Since some *Trichoderma* species are naturally resistant to fungicides, they can be combined in a single mixture. In a field trial of dry bean production, *T*. *virens* and thiophanate-methyl were discovered together in *Fusarium solani* and *Fusarium oxysporum* [68]. Similar results were recorded for the treatment of *Rosellinia necatrix*-induced avocado white rot, where the application of *Trichoderma* species combined with a low dose of fluazinam proved to be more effective than either treatment alone [69]. Moreover, a low dose of the broad-spectrum fungicide tolclofos-methyl combined with *Trichoderma* spp. was superior to the fungicide alone despite *Trichoderma* spp. not being effective against *Acremonium strictum* and *F*. *oxysporum* in an *in vitro* experiment [70]. A parallel study found that thiabendazole mixed with *Cryptococcus laurentii* at 10% of the recommended dose had the longest-lasting and strongest effects on controlling *B*. *cinerea*, an important postharvest disease, on stored apples [71]. The combination proved even more effective and lasted longer than the biocontrol yeast alone in controlling a thiabendazole-resistant isolate of Botrytis cinerea on apples that had been harvested and treated with newer fungicides. More effective than the treatment alone was a combination of the biocontrol yeasts (*C*. *laurentii* or *Rhodotorula kratochvilovae*) with a small dose of either cyprodinil or boscalid [72]. Like fungal antagonists, the main advantage of bacterial antagonists is enhanced in disease management against soilborne diseases. For instance, tomato disease treatment with *Bacillus megaterium* against *F*. *oxysporum* and a small dose of the fungicide carbendazim in plant packs should improve the situation [73]. Full disease control was achieved by the combination, even outperforming the administration of the fungicide at a dose that was ten times greater. Comparable results were obtained in the same setup by employing a combination of *Pseudomonas fluorescens* and a tenfold lower dose of benomyl, which reduced the disease as much as using the fungicide at its full dose alone [74]. *Bacillus subtilis* combined with azoxystrobin produced the maximum yield on zucchini and the best disease control against powdery mildew (induced by *Podosphaera xanthii*) in multiple greenhouse experiments [75]. A list of microbial biofungicides used as biological control agents for fungal plant diseases is indicated in Table 1.

### 2.5. Current Perspectives and Future Research Directions

Microbial biofungicides are a great alternative to chemical pesticides for farmers who need to protect their plant crops. However, there is very little demand for and supply of microbial biofungicides, which discourages both producers and users. If we look at the current research status from collected databases, top priorities are reflected more for academic purposes than for product development. The majority of papers focus on the screening tests, the methodology of evaluation for the biological activity, and the biochemical mechanisms of action. Before becoming an economically feasible alternative to chemical control, biopesticides must satisfy several requirements which must be considered as a whole an effective microbial strain showing a reliable effectiveness must be selected. A technology providing high-quality biomass and adequate formulation must be developed. A knowledge of the ecological requirements for survival, colonization, and/or biological control activity is required. The toxicological confidence of the user safety and the ecotoxicological safety must be controlled. The production and availability of microbial biofungicides can also be improved by providing grants or capital to researchers, business owners, producers, and marketers. Microbial biofungicides continue to face numerous difficulties in their manufacture, application, and development. Therefore, further investigation into the mechanisms that increase the stability and shelf life of microbial biofungicides will greatly contribute to boosting their efficacy. In addition, there are still issues that need to be resolved regarding standardization and field-scale microbial performance tests. To enable the commercialization of microbial biofungicides, additional production, delivery, and formulation research is needed. The mixing of the public and private sectors could advance the development, study, and distribution of environmentally friendly fungicides in underdeveloped countries. In addition, more funding for commercial investors, public-funded programs, and biofungicide companies is required. Creating strict regulatory frameworks to keep microbial biofungicides accessible at reasonable costs in developing nations is a crucial issue.

## 3. Concluding Remarks

Synthetic fungicides are primarily used by farmers all over the world to manage infections in their agricultural ecology. However, since the fact that these fungicides pose a hazardous effect on humans and the environment, it became essential to design a suitable pathogen control strategy, such as the application of microbial biofungicides. These microbial biofungicides are eco-friendly and safer and play an important role in modern agriculture. Microbial biopesticides give eco-friendly alternatives to synthetic pesticides, yet they confront various difficulties in their production, formulation, and application. It appears to be that microbial biopesticides will have a more extensive use in the future as their application techniques enhance as less expensive inert materials are recognized for different formulations. Microbial biofungicides offer a more balanced plant protection product application, and in the future formulation, products should have more balance between production cost and efficiency. Development related to the formulation type would possibly shift from a single microorganism-based product to a microbial consortium-based formulation. Significant advancement has been made in the production of new formulation products and application methods; however, there is still much work to be performed. For further research to improve production and application techniques, scientists and researchers are likely to provide safe and effective products for plant disease management. Moreover, the advancement of methodologies and multidisciplinary research will be the focus of future studies to produce high-quality, secure, and cost-efficient plant protection solutions.

## Figures and Tables

**Table 1:** Microbial biofungicides used as biocontrol agents for fungal plant disease.

Phytopathogens	Hosts	Diseases	Biocontrol agents	References
*Botrytis cinerea*	Apple	Gray mold	*Metschnikowia pulcherrima*	Fernandez-San Millan et al. [76]
*Botrytis cinerea* and *Penicillium italicum*	Apple	Blue mold	*Pichia membranifaciens* and *Wickerhamomyces anomalus*	Błaszczyk et al. [77]
*Botrytis cinerea*	Grapes and apples	Gray mold	*Meyerozyma guilliermondii* and *Aureobasidium pullulans*	Sepúlveda et al. [78]
*Botrytis cinerea* and *Penicillium expansum*	Apple	Apple rot	*Aureobasidium subglaciale*	Zajc et al. [79]
*Botryosphaeria dothidea*	Apple	Ring rot	*Serratia plymuthica*	Sun et al. [80]
*Colletotrichum gloeosporioides*	Avocado	Anthracnose	*Yamadazyma mexicana*	González-Gutiérrez et al. [81]
*Penicillium digitatum*	Citrus	Green mold	*Lactobacillus plantarum and Lactobacillus plantarum*	Chen et al. [82]
*Penicillium digitatum*	Mandarin fruits	Green mold	*Debaryomyces hansenii*, *Lactobacillus plantarum*, *Metschnikowia pulcherrima*, *Pichia guilliermondii*, and *Rhodotorula minuta*	Bhan et al. [83]
*Penicillium digitatum*	Citrus	Green mold	*Pichia kudriavzevii*	Delali et al. [84]
*Fusarium solani and Fusarium oxysporum*	Dry bean	Wilt diseases	*Trichoderma* spp.	Abd-El-Khair et al. [68]
*Botrytis cinerea*	Grapes	Gray mold	*Bacillus amyloliquefaciens*	Zhou et al. [85]
*Botrytis cinerea*	Grapes	Gray mold	*Lactobacillus plantarum*	Chen et al. [86]
*Pestalotiopsis theae*	Tea plants	Gray blight	*Paecilomyces lilacinus*	Wang et al. [87]
*Plasmodiophora brassicae*	Pak choi	Clubroot	*Trichoderma viride*	Arif et al. [88]
*Colletotrichum gloeosporioides*	Mango	Anthracnose	*Penicillium citrinum*	Sandy et al. [89]
*Colletotrichum gloeosporioides*	Mango	Anthracnose	*Lactobacillus acidophilus*	Fenta and Kibret [90]
*Colletotrichum gloeosporioides*	Mango	Anthracnose	*Streptomyces* sp.	Zhou et al. [91]
*Colletotrichum gloeosporioides*	Mango	Anthracnose	*Bacillus amyloliquefaciens*	Liang et al. [92]
*Penicillium digitatum* and *Penicillium italicum*	Orange and lemon	Green mold and blue mold	*Bacillus amyloliquefaciens*, *Bacillus pumilus*, and *Bacillus subtilis*	Hammami et al. [93]
*Penicillium digitatum*, *Penicillium italicum*, and *Geotrichum citri-aurantii*	Lemon	Green mold	*Clavispora lusitaniae*	Pereyra et al. [94]
		Blue mold		
*Aspergillus flavus*	Rice	Rice mold	*Bacillus velezensis*	Li et al. [95]
*Penicillium digitatum*	Orange	Green mold	*Bacillus* sp.	Tian et al. [96]
*Penicillium italicum*	Orange	Blue mold	*Pseudomonas fluorescens*	Wang et al. [97]
*Aspergillus flavus*	Peanuts	Mycotoxins	*Bacillus subtilis*	Ling et al. [98]
*Colletotrichum* spp., and *Fusarium* sp.	Chili pepper	Fruit rot	*Streptomyces tuirus*	Renuka et al. [99]
*Colletotrichum gloeosporioides*	Chili pepper	Anthracnose	*Trichoderma koningiopsis*	Ruangwong et al. [100]
*Colletotrichum truncatum*	Chili pepper	Anthracnose	*Trichoderma asperellum and Trichoderma harzianum*	Yadav et al. [101]
*Fusarium oxysporum*	Potato	Fusarium rot	*Bacillus* sp.	Ntemafack et al. [102]
*Aspergillus flavus*	Tomato	Spoilage	*Aureobasidium pullulans*	Podgórska-Kryszczuk [103]
*Alternaria arborescens*	Tomato	Tomato rot	*Torulaspora indica*	Bosqueiro et al. [104]
*Botrytis cinerea*	Tomato	Gray mold	*Trichoderma harzianum*	Imran et al. [105]
*Botrytis cinerea*	Strawberry	Gray mold	*Lactobacillus plantarum*	Chen et al. [106]
*Colletotrichum gloeosporioides*	Strawberry	Anthracnose	*Bacillus amyloliquefaciens*	Wu et al. [107]
*Colletotrichum gloeosporioides*	Strawberry	Anthracnose	*Trichoderma asperellum*	El Kaissoumi et al. [108]
*Botrytis cinerea*	Vineyard	Gray mold	*Metschnikowia pulcherrima* and *Aureobasidium pullulans*	Agarbati et al. [109]

## Data Availability

No data were used to support the study.

## Abbreviations

BCAs: Biocontrol agents
CFU: Colony-forming unit
MRL: Maximum residue level
IDM: Integrated disease management.
